# ﻿*Violaxinchengensis* (Violaceae), a new species from central Guangxi, China

**DOI:** 10.3897/phytokeys.253.128972

**Published:** 2025-03-04

**Authors:** Gui-Yuan Wei, Chuan-Gui Xu, Ying-Jing Li, Bin Feng, Qi-Min Hu, Chao Yang, Xin-Cheng Qu, You Nong

**Affiliations:** 1 Guangxi Key Laboratory of Traditional Chinese Medicine Quality Standards; Guangxi Institute of Chinese Medicine & Pharmaceutical Science, No. 20–1 Dongge Road, Nanning, Guangxi, China Guangxi Institute of Chinese Medicine & Pharmaceutical Science Nanning China

**Keywords:** Morphology, new species, sinkhole, taxonomy, *Viola*

## Abstract

*Violaxinchengensis* (Violaceae), a new species from Guangxi, China, is established on the basis of morphological and molecular evidence. This new species resembles *V.lucens*, but differs from the latter by its stipules margin long fimbriate-dentate (vs. fimbriate-dentate), stolon absent (vs. stolon slender, often producing a new plant at the top) and sepals 4–6 mm, glabrous (vs. 2.5–3 mm, villous). *Violaxinchengensis* is similar to *V.fargesii*, but it can be easily distinguished by its pedicels sparsely white villous (vs. densely spreading white puberulous), stolon absent (vs. stolon longer, elongated, puberulous, sometimes stem-like) and sepals 4–6 mm, glabrous (vs. 7–9 mm, puberulous). Our morphology analysis confirms that the new species belongs to V.sect.Plagiostigmasubsect.Diffusae. Photographs, an illustration, a distribution map and comparisons with the most similar species are also provided.

## ﻿Introduction

*Viola* L. is the largest genus of the family Violaceae, with approximately 664 species that are classified in two subgenera, 31 sections and 20 subsections around the world (Marcussen et al. 2022). This genus has a high level of morphological differentiation and there are hybridisation and horizontal evolution amongst its sections and species (Marcussen et al. 2015). However, the delimitation of the species with stolons distributed in southern and south-western China remains highly problematic and new species are still being discovered (Zhou and Xing 2007; Chen and Yang 2009; Dong et al. 2009; Ning et al. 2012; Huang et al. 2021; Li et al. 2022; Huang et al. 2023a; Huang et al. 2023b).

Guangxi is located in the southwest of China and is a biodiversity hotspot where many new species or new species records have been recently found (Hu et al. 2019; Luo et al. 2020; Feng et al. 2021; Huang et al. 2022; Nong et al. 2023; Nong et al. 2024). During our field surveys in Xincheng County, Guangxi in April 2024, we found a special *Viola* population in flowers and fruits that was morphologically similar to the species *V.fargesii* H. Boissieu and *V.lucens* W. Becker in having rhizomes erect, leaves basal, stipules margin fimbriate-dentate and ovaries glabrous. After careful comparisons and verifications, we carried out one more field survey to confirm that the unusual plant is a species of *Viola* new to science and we describe it below. Photographs, an illustration, a distribution map and a table of comparisons with the most similar species are also provided.

## ﻿Materials and methods

### ﻿Morphology

The new species was described, based on field observations made in April 2024 and examination of herbarium specimens. Other related *Viola* species were examined, based on online images from the Kew Herbarium Catalogue (http://apps.kew.org/herbcat/gotoHomePage.do) and JSTOR Global Plants (http://plants.jstor.org/) and specimens from GXMI. We also observed living plants of the new species at flowering and fruiting time (April and May). We observed characters of stems, leaves, pedicels, flowers, receptacles, petals, stamens, gynoecium and capsule.

Descriptions were based on observations from herbarium specimens. Measurements were made with a tape measure and calipers. The structure of the indumentum and its distribution were observed and described under a dissecting microscope at magnifications of more than 20×. Additional information on locality, habitat, plant form and fruits was collected in the field and taken from herbarium labels. We followed the IUCN Categories and Criteria (IUCN 2022) to assess the provisional conservation status of the new species.

### ﻿Molecular phylogenetic analysis

Leaf material of the putative new species was collected and stored with silica gel in zip-lock plastic bags until use for comparisons and taxonomical treatment. In this study, molecular phylogenetic analysis, based on the ITS dataset, was firstly conducted to resolve the phylogenetic position of the new species. Genomic DNA of the potential new species was extracted from silica-gel-dried leaves using the modified 2× CTAB procedure of Doyle and Doyle (1987). Primers used for the polymerase chain reaction (PCR) amplification and sequencing were the same as those of Chen et al. (2021), while PCR procedures followed those described in Chen et al. (2016). Another 42 sample sequences were obtained from NCBI (Gong et al. 2010; Liang and Xing 2010). The specimen information of samples and GenBank accession numbers for all sequences are listed in Table 1.

**Table 1.** Vouchers of specimens and GenBank accession number.

Accession no.	Taxon	Voucher
EF660538.1	*Melicytuschathamicus*	–
JQ950556.1	*Violaalbida*	Fengcheng, Liaoning, Chen Y. S. 01819036 (PE)
JF830900.1	*Violaamamiana*	–
OQ848672.1	*Violaaustroyunnanensis*	–
MN493162.1	*Violabaoshanensis*	Hunan, Liu W. SYS00142785 (SYS)
LC669903.1	*Violabetonicifolia*	Jingxi, Guangxi, Qin H. N. 01990960 (PE)
DQ787768.1	*Violachaerophylloides*	Fengcheng, Liaoning, Chen Y. S. 01840427 (PE)
OP935155.1	*Violachangii*	Guangdong, Liang G. X. 0765177 (IBSC)
JQ950563.1	*Violadactyloides*	Daxinganling, Heilongjiang, Chen Y. S. 01840253 (PE)
MH711664.1	*Violadavidii*	Leishan, Guizhou, Chen Y. S. 01840420 (PE)
MH711723.1	*Violadiffusa*	Leibo, Sichuan, He M. Y. 02093842 (PE)
FJ002914.1	*Violadiffusoides*	Sichuan, Y.C.Yang 00025459 (PE)
JQ950564.1	*Violadissecta*	Zhenan, Shanxi, Zhang C. F. 02247331 (PE)
JQ950567.1	*Violaeizanensis*	Janpan, Miyoshi Furuse 01207914 (PE)
AY928297.1	*Violahirtipes*	Tonghua, Jilin, Chen Y. S. 01840415 (PE)
AY928295.1	*Violajaponica*	Pengzhe, Jiangxi, Qin H. N. 01861607 (PE)
MT923897.1	*Violakunawarensis*	Hejing, Xinjiang, Chen Y. S. 02038258 (PE)
FJ002913.1	*Violalucens*	Lechang, Guangdong, Chen Y. S. 01840441 (PE)
OR483796.1	*Violananlingensis*	Nanling, Guangdong, Wang G. F. 0765184 (IBSC)
AY928298.1	*Violapatrinii*	Hengren, Liaoning, Chen Y. S. 01840394 (PE)
MH710789.1	*Violaphalacrocarpa*	Taian, Shandong, Chen Y. S. 01861292 (PE)
MH711011.1	*Violaphilippica*	Fangshan, Beijing, Shi L. 02112316 (PE)
JQ950572.1	*Violapinnata*	Beijing, Wang J. W. PEY0004742 (PEY)
AY928279.1	*Violaraddeana*	Janpan, Miyoshi Furuse 01220858 (PE)
AY928301.1	*Violaseoulensis*	Korea, G.-N.Jeon,B.-S.Kim 020407329 (PE)
DQ787772.1	*Violasieboldiana*	–
AB828325.1	*Violasieboldii*	Janpan, Miyoshi Furuse 00159231 (PE)
FJ002912.1	*Violatriangulifolia*	Lingui, Guangxi, Liu B. 01990939 (PE)
KC330744.1	*Violavariegata*	Tonghua, Jilin, Chen Y. S. 01840188 (PE)
AY928283.1	*Violaverecunda*	Xingan, Guangxi, Chen Y. S. 01819105(PE)
AY928308.1	*Violaviolacea*	Jiujiang, Jiangxi, Chen Y. S. 01840530 (PE)
AY928291.1	*Violawoosanensis*	–
PV089292	*Violaxinchengensis*	Xincheng, Guangxi, Nong Y. 051188 (GXMI)
FJ002915.1	*Violayunnanensis*	Lingshui, Hainan, Chen Y. S. 01819675 (PE)

All sequences were assembled and edited using Geneious v.7.06 (Kearse et al. 2012) and then aligned using MUSCLE (Edgar 2004) and manually adjusted in MEGA 6.0 (Tamura et al. 2013). Bayesian Inference (BI) (Ronquist et al. 2012) and Maximum Likelihood (ML) (Stamatakis 2014) analyses were used for phylogenetic reconstruction and detailed settings for the two analyses followed those described in Chen et al. (2021). Phylogenetic construction was conducted by Maximum Likelihood with MEGA 6.0 (Tamura et al. 2013), selecting the best-fit model of Jukes-Cantor with 2000 bootstraps. The resulting trees with posterior probabilities (PP) and Bootstrap support (BS) values were visualised and annotated in TreeGraph 2 (Stöver and Müller 2010). Topological incongruence between the two reconstructions was visually inspected, based on the thresholds of PP ≥ 0.95 and/or BS ≥ 70%. After excluding the taxa that exhibited strong conflicts between the nuclear tree and the plastid tree, the combined nuclear dataset and the combined plastid dataset were then concatenated for phylogenetic analyses. *Melicytuschathamicus* (F.Muell.) Garn.-Jones. was used as outgroup.

## ﻿Results and discussion

**Figure 1.**
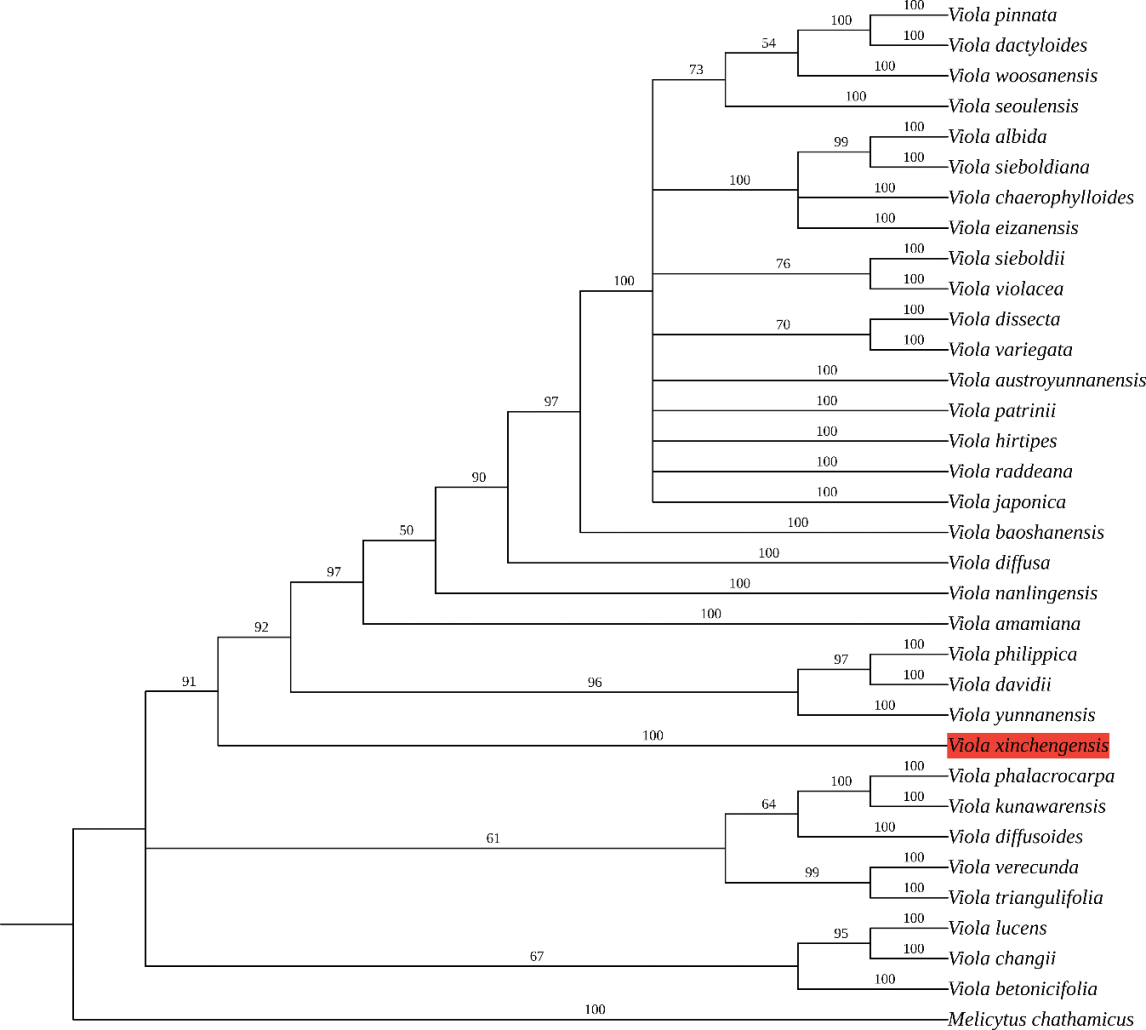
ML tree of the new species *Violaxinchengensis* sp. nov. and its related species, based on the ITS dataset. Bootstrap values of the Maximum Likelihood are shown along the branches.

The ITS dataset comprises 34 accessions representing 34 species, including the outgroup (Table 1). The aligned matrix of ITS sequences was 656 bp in total. The result of ML is shown in Fig. 1. The samples of the putative new species (red background) clustered into a strongly supported monophyletic lineage, forming a weak sister relationship with a clade composed of *V.yunnanensis*, *V.davidii* and *V.philippica*. Based on morphological characters and phylogenetic results, we recognise this unfamiliar violet as a distinct species and describe it here as *V.xinchengensis* Y.Nong & G.Y.Wei.

### ﻿Taxonomic treatment

#### ﻿ Viola xinchengensis

Taxon classificationPlantaeMalpighialesViolaceae

Y.Nong & G.Y.Wei sp. nov.

BD86D211-828D-5485-A0C4-5146FC62FC3C

urn:lsid:ipni.org:names:77357572-1

Figs 1–4

##### Chinese name.

xīn chéng jǐn cài (忻城堇菜).

##### Diagnosis.

*Violaxinchengensis* is most similar to *V.lucens*, but differs from the latter by its stipules margin long fimbriate-dentate (vs. fimbriate-dentate), stolon absent (vs. stolon slender, often producing a new plant at the top) and sepals 4–6 mm, glabrous (vs. 2.5–3 mm, villous). *Violaxinchengensis* is similar to *V.fargesii*, but it can be easily distinguished by its pedicels sparsely white villous (vs. densely spreading white puberulous) and sepals 4–6 mm, glabrous (vs. 7–9 mm, puberulous). More detailed morphological differences amongst the three similar species are shown in Table 2.

**Table 2.** Main morphological differences amongst *Violaxinchengensis*, *V.lucens*, and *V.fargesii*.

Morphological traits	*Violaxinchengensis*	*V.lucens*	*V.fargesii*
Stolon	absent	slender, often producing new plant at top	longer, elongated, puberulous, sometimes stem-like
Stipules	margin long fimbriate-dentate	margin fimbriate-dentate	margin long fimbriate-dentate
Petiole	villous, narrowly winged only in upper part	densely villous, wingless	densely villous, wingless
Leaf blade	ovate, 1.5–2.5 cm × 1.5–2 cm, base cordate	oblong-ovate, ovate or oblong, 1–2(–3) × 0.5–1.3 cm, base cordate or rounded	ovate or broadly ovate, sometimes suborbicular, 2–6 × 2–4.5 cm, base shallowly cordate
Flowers	purplish	light bluish violet	white
pedicels	sparsely white villous	sparsely puberulous	densely spreading white puberulous
Sepals	narrowly ovate-lanceolate or lanceolate, 4–6 mm, glabrous	narrowly lanceolate, 2.5–3 mm, villous	narrowly ovate-lanceolate or lanceolate, 7–9 mm, puberulous
Petals	oblong-obovate, 6–10 mm, lateral ones bearded	narrowly lanceolate, 2.5–3 × ca. 1 mm, lateral ones glabrous	oblong-obovate, 1–1.5 cm, lateral ones slightly bearded
Spur	1.5–2 mm	ca. 1.5 mm	2–2.5 mm
Ovary	ovoid, glabrous	globose, glabrous	conic, glabrous
Styles	base slightly geniculate, slightly flat at apex, conspicuously margined on lateral sides, shortly beaked in front, with a stigma hole open upwards at tip of beak	base geniculate, thickened at apex; stigmas narrowly margined on lateral sides, apex shortly beaked	base slightly geniculate, slightly flat at apex, conspicuously margined on lateral sides, shortly beaked in front, with a stigma hole open upwards at tip of beak
Capsule	narrowly orbicular, 5 mm, glabrous	ovoid-orbicular, 5 mm, glabrous	narrowly orbicular, 8 mm, glabrous

**Figure 2.**
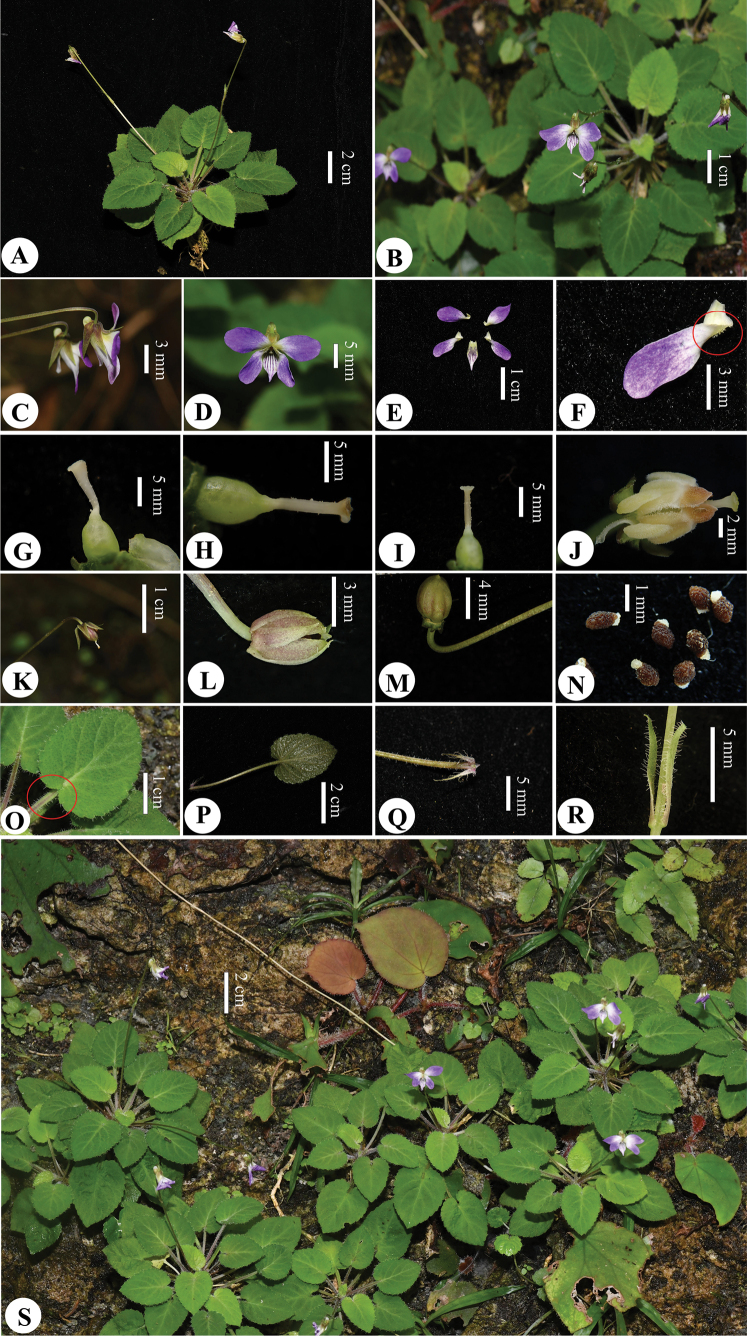
*Violaxinchengensis* Y.Nong & G.Y.Wei **A** plant (top view) **B** plant (flowering) **C** flowers (lateral view) **D** flower (front view) **E** petals **F** lateral petal **G–I** ovary and style **J** stamens **K–M** capsule **N** seeds **O** leaf (adaxial surface) **P** leaf (abaxial surface) **Q** stipules **R** bract **S** habitat (Photographed and edited by You Nong).

##### Type.

China • Guangxi: Xincheng, 23°59'42"N, 108°44'28"E, alt. 370 m, on the cliff at the bottom of a sinkhole, 20 April 2024, *Y. Nong NY2024042002* (holotype GXMI! 051188; isotypes IBK!).

**Figure 3.**
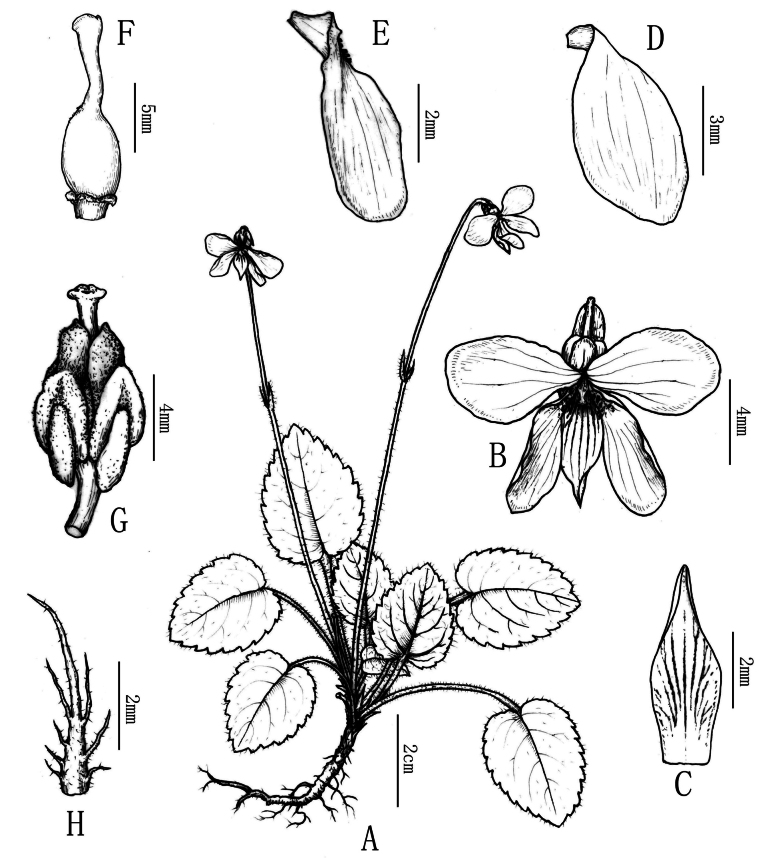
Line drawing of *Violaxinchengensis* Y.Nong & G.Y.Wei **A** flowering plant **B** flower **C** anterior petal **D** upper petal **E** lateral petal **F** ovary and style **G** stamens and pistil **H** stipule. Drawn by Xin-cheng Qu.

##### Description.

Perennial herbs, small, 5–10 cm tall; rhizome erect, sometimes elongate, with short internodes, ca. 2 mm, lateral stem and stolon absent. Leaves nearly basal; stipules adnate to petioles for about 1/8 at base, brown, lanceolate, 5–7 mm × 1–2 mm, margin long fimbriate-dentate, apex acuminate. Petiole 1.5–3.5 cm, villous, narrowly winged only in the upper part. Leaf blade ovate, 1.5–2.5 cm × 1.5–2 cm, apex acute, base cordate, margin crenate, both surfaces densely villous. Pedicels much exceeding the leaves, glabrous or sparsely villous, 2-bracteolate above middle; bracteoles opposite, linear, 6–8 mm, margin villous. Sepals ovate-lanceolate, entire, 4–6 mm, apex acuminate, basal auricles short, ca. 2 mm, glabrous. Flower 1.0–1.5 cm in diameter, petals 5, white with purple or purplish, posterior and lateral ones oblong-obovate, ca. 7–8 mm × 3–5 mm, narrow at the base, lateral petals white with purple, lateral ones bearded, anterior one boat-shaped, 5–8 mm (spur included); spur saccate, short, 1.5–2 mm, ca. 2 mm in diam. Anthers ca. 1.2 mm, connective appendages ca. 0.5 mm; nectariferous glands broadly triangular, ca. 0.5 mm. Ovary ovoid, glabrous; style clavate, base slightly geniculate, thickened upwards, slightly flat at the apex, conspicuously margined on lateral sides, shortly beaked in front, with a stigma hole open upwards at the tip of the beak. Cleistogamous flowers ca 1.5 mm long; bracteoles linear, 6–8 mm, margin villous, acuminate at the apex. Sepals green, entire, 4–6 mm, apex acuminate. Petal 1, ovate, white with purple, 1.0–1.5 cm long. Capsule ovoid, dehiscence explosive, ca. 5 mm, glabrous. Seeds brown, ovoid, 1 mm, densely covered with tubercles.

**Figure 4.**
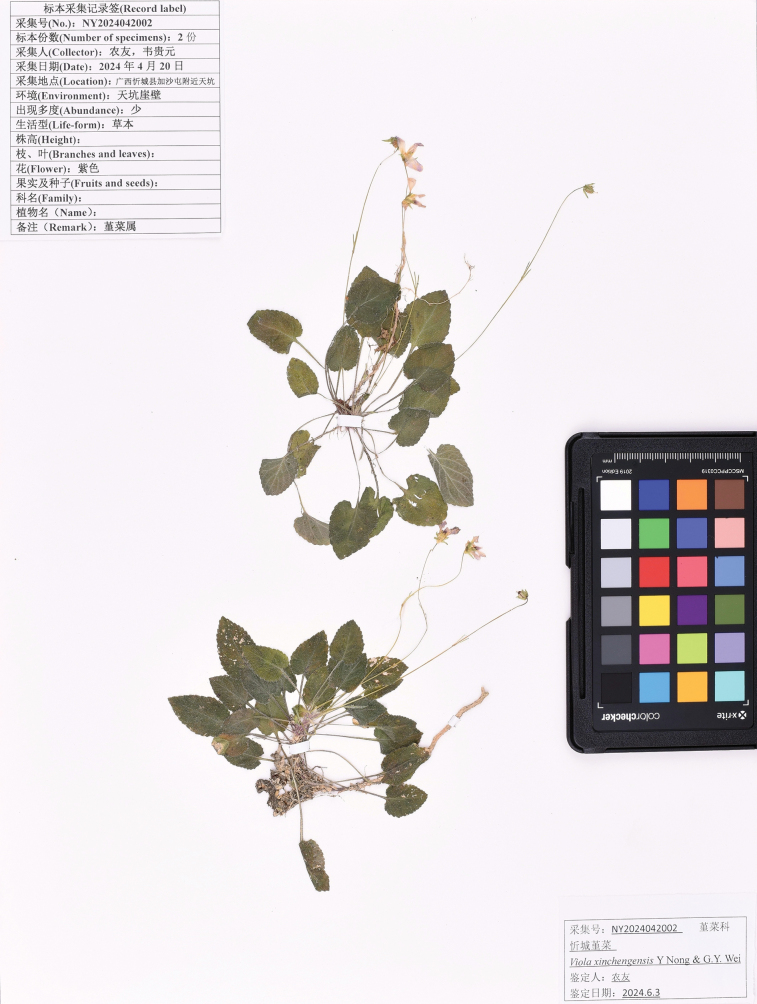
The holotype specimen of *Violaxinchengensis* Y.Nong & G.Y.Wei.

##### Phenology.

Flowering and fruiting from April to June.

##### Etymology.

The specific epithet “xinchengensis” refers to the type locality, Xincheng County (忻城县), which is situated in central Guangxi, southwest China.

##### Distribution and habitat.

**Figure 5.**
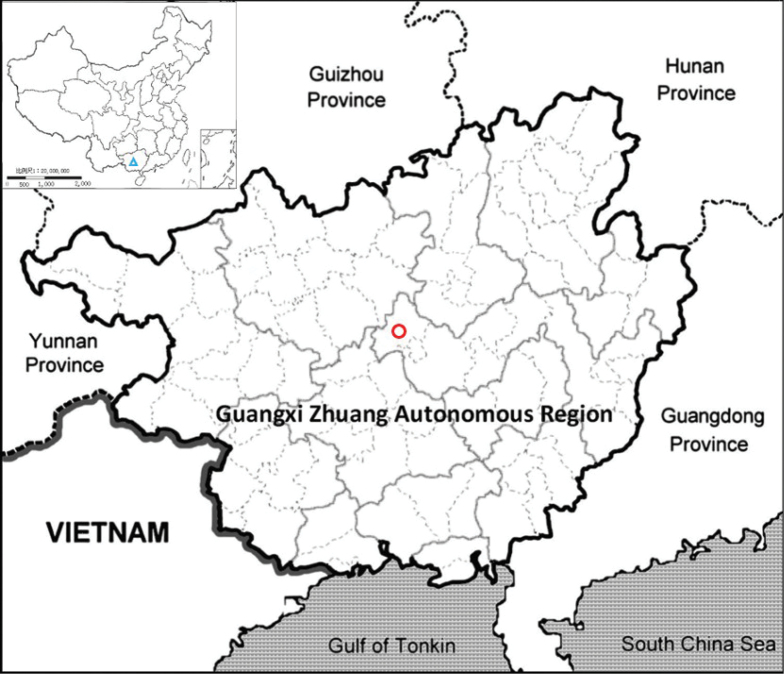
The distribution of *Violaxinchengensis* Y.Nong & G.Y.Wei (red circle) in Guangxi (blue triangle in insert map), China.

The new species is known only from central Guangxi, China (Fig. 5). It has been found mainly on the cliff at the bottom of a sinkhole at elevations of 370 m. It usually grows with *Begoniapseudoleprosa* C. I Peng & al. and *Primulinasclerophylla* (W. T. Wang) Yan Liu on the damp cliffs.

##### IUCN Red List Category.

Data available for the new species are still insufficient to assess its conservation status. According to the IUCN Criteria (IUCN 2022), it is considered Data Deficient (DD) until more information becomes available. Although *Violaxinchengensis* currently has relatively good growth, further collection and monitoring are necessary to allow more conclusive estimations about the rarity and vulnerability of the species.

##### Additional specimens examined (paratypes).

China • Guangxi: Xincheng, 23°59'42"N, 108°44'28"E, alt. 370 m, at the bottom of a sinkhole, 26 April 2024 *Y. Nong NY20240042602* (GXMI) • Xincheng, 23°59'42"N, 108°44'28"E, alt. 370 m, at the bottom of a sinkhole, 12 June 2024 *G. Y. Wei WGY20240061201* (GXMI).

##### Taxonomic notes.

*Violaxinchengensis* lacks bulbils, lateral stems and stolons. Stipules are adnated to petioles for about 1/8 at base, membranous, glandular-lacerate. Bottom petal is 7–12 mm long, including the spur. Style apex margined and flattened. According to the study of *Viola* (Marcussen et al. 2022), *V.xinchengensis* belongs to V.sect.Plagiostigmasubsect.Diffusae W. Becker.

## Supplementary Material

XML Treatment for Viola xinchengensis

## ﻿Additional information

### Author ORCIDs

Gui-Yuan Wei https://orcid.org/0000-0003-0652-1213

Chuan-Gui Xu https://orcid.org/0009-0000-6263-3821

Ying-Jing Li https://orcid.org/0009-0008-4849-2426

Bin Feng https://orcid.org/0000-0003-0387-3635

Qi-Min Hu https://orcid.org/0009-0003-0490-3557

Chao Yang https://orcid.org/0009-0004-9458-4007

Xin-Cheng Qu https://orcid.org/0009-0009-9078-9976

You Nong https://orcid.org/0000-0001-7004-0946

### Data availability

All of the data that support the findings of this study are available in the main text.

## References

[B1] Chen YS Yang QE (2009) Two new stoloniferous species of *Viola* (Violaceae) from China. Botanical Journal of the Linnean Society 159(2): 349–356. 10.1111/j.1095-8339.2008.00911.x

[B2] Chen YP Drew BT Li B Soltis DE Soltis PS Xiang CL (2016) Resolving the phylogenetic position of *Ombrocharis* (Lamiaceae), with reference to the molecular phylogeny of tribe Elsholtzieae. Taxon 65(1): 123–136. 10.12705/651.8

[B3] Chen YP Liu A Yu XL Xiang CL (2021) A preliminary phylogenetic study of *Paraphlomis* (Lamiaceae) based on molecular and morphological evidence. Plant Diversity 43(3): 206–215. 10.1016/j.pld.2021.03.002 34195505 PMC8233522

[B4] Dong AQ Zhou JS Gong Q (2009) A new species of *Viola* (Violaceae) from Guangdong, China. Novon 19(4): 457–460. 10.3417/2007157

[B5] Doyle JJ Doyle JL (1987) A rapid DNA isolation procedure for small quantities of fresh leaf tissue. Phytochemical Bulletin 19: 11–15. https://cir.nii.ac.jp/crid/1572543024016000128?lang=en

[B6] Edgar RC (2004) MUSCLE: Multiple sequence alignment with high accuracy and high throughput. Nucleic Acids Research 32(5): 1792–1797. 10.1093/nar/gkh340 15034147 PMC390337

[B7] Feng XX Xiao Y Liu ZX Li RK Wei D Tian DK (2021) *Begoniapseudoedulis*, a new species in Begoniasect.Platycentrum (Begoniaceae) from southern Guangxi of China. PhytoKeys 182: 113–124. 10.3897/phytokeys.182.69074 34720624 PMC8516824

[B8] Gong Q Zhou JS Zhang YX Liang GX Chen HF Xing FW (2010) Molecular systematics of genus *Viola* L. in China. Redai Yaredai Zhiwu Xuebao 18(6): 633–642. 10.3969/j.issn.1005-3395.2010.06.007

[B9] Hu R Wei S Liufu Y Nong Y Fang W (2019) *Camelliadebaoensis* (Theaceae), a new species of yellow camellia from limestone karsts in southwestern China. PhytoKeys 135: 49–58. 10.3897/phytokeys.135.38756 31824208 PMC6895178

[B10] Huang YS Kang N Zhong XJ Liao WB Fan Q (2021) A new species of *Viola* (Violaceae) from Guangdong Province, China. PhytoKeys 176: 67–76. 10.3897/phytokeys.176.65443 33958940 PMC8065024

[B11] Huang YS Nong SY Li XK Xie G Tong YH (2022) *Vacciniumbangliangense*, a new species of Ericaceae from limestone areas in Guangxi, China. PhytoKeys 194: 23–31. 10.3897/phytokeys.194.81018 35586320 PMC9016033

[B12] Huang YS Ding JH Ye QT Dai JM Zhong ZM Fan Q (2023a) Four new species of *Viola* (Violaceae) from southern China. Nordic Journal of Botany 2023(6): e03941. 10.1111/njb.03941

[B13] Huang YS Jia XY Zeng QJ Wen WL Fan Q (2023b) *Violapendulipes* (Violaceae), a new species from Guangdong Province, China. Nordic Journal of Botany 2023(12): e04165. 10.1111/njb.04165

[B14] IUCN (2022) Guidelines for using the IUCN Red List categories and criteria, version 14. Prepared by the Standards and Petitions Committee. https://www.iucnredlist.org/resources/redlistguidelines [Accessed 6 May 2024]

[B15] Kearse M Moir R Wilson A Stones-Havas S Cheung M Sturrock S Buxton S Cooper A Markowitz S Duran C Thierer T Ashton B Meintjes P Drummond A (2012) Geneious Basic: an integrated and extendable desktop software platform for the organization and analysis of sequence data. Bioinformatics 28(12): 1647–1649. 10.1093/bioinformatics/bts199 22543367 PMC3371832

[B16] Li XC Wang ZW Wang Q Ge BJ Chen B Yu P Zhong X (2022) *Violashiweii*, a new species of *Viola* (Violaceae) from karst forest in Guizhou, China. PhytoKeys 196: 63–89. 10.3897/phytokeys.196.83176 36762027 PMC9848997

[B17] Liang GX Xing FW (2010) Infrageneric phylogeny of the genus *Viola* (Violaceae) Based on trnL-trnF, psbA-trnH, rpL16, ITS Sequences, Cytological and Morphological Data. Acta Phytoecologica Sinica 32(6): 477–488.

[B18] Luo YJ Ni SD Jiang Q Huang BG Liu Y Huang YS (2020) *Aristolochiayachangensis*, a new species of Aristolochiaceae from limestone areas in Guangxi, China. PhytoKeys 153: 49–61. 10.3897/phytokeys.153.52796 32765180 PMC7381434

[B19] Marcussen T Heier L Brysting A Oxelman B Jakobsen K (2015) From gene trees to a dated allopolyploid network: Insights from the angiosperm genus *Viola* (Violaceae). Systematic Biology 64(1): 84–101. 10.1093/sysbio/syu071 25281848 PMC4265142

[B20] Marcussen T Ballard HE Danihelka J Flores AR Nicola MV Watson JM (2022) A revised phylogenetic classification for *Viola* (Violaceae). Plants 11(17): e2224. 10.3390/plants11172224 PMC9460890 36079606

[B21] Ning ZL Zeng ZX Chen L Xu BQ Liao JP (2012) *Violajinggangshanensis* (Violaceae), a new species from Jiangxi, China. Annales Botanici Fennici 49(5): 383–386. 10.5735/085.049.0610

[B22] Nong Y Xu CG Wei GY Yan KJ Qu XC Zhang ZJ Hu RC Huang YF (2023) *Walsuraguangxiensis* (Meliaceae), a new species from Guangxi, China. PhytoKeys 234: 219–227. 10.3897/phytokeys.234.106205 37927972 PMC10625162

[B23] Nong Y Lai KD Qin YR Wei GY Yan KJ Xu CG Zhao ZY Hu RC Huang YF (2024) *Aletrisguangxiensis* (Nartheciaceae), a new species from Guangxi, China. PhytoKeys 237: 79–89. 10.3897/phytokeys.237.115037 38282985 PMC10819618

[B24] Ronquist F Teslenko M van der Mark P Ayres DL Darling A Höhna S Larget B Liu L Suchard MA Huelsenbeck JP (2012) MrBayes 3.2: Efficient Bayesian phylogenetic inference and model choice across a large model space. Systematic Biology 61(3): 539–542. 10.1093/sysbio/sys029 22357727 PMC3329765

[B25] Stamatakis A (2014) RAxML version 8: A tool for phylogenetic analysis and post-analysis of large phylogenies. Bioinformatics (Oxford, England) 30(9): 1312–1313. 10.1093/bioinformatics/btu033 24451623 PMC3998144

[B26] Stöver BK Müller KF (2010) TreeGraph 2: Combining and visualizing evidence from different phylogenetic analyses. BMC Bioinformatics 11(1): 1–7. 10.1186/1471-2105-11-7 20051126 PMC2806359

[B27] Tamura K Stecher G Peterson D Filipski A Kumar S (2013) MEGA6: Molecular evolution-ary genetics analysis version 6.0. Molecular Biology and Evolution 30(12): 2725–2729. 10.1093/molbev/mst197 24132122 PMC3840312

[B28] Zhou JS Xing FW (2007) *Violachangii* sp. nov. (Violaceae) from Guangdong, southern China. Nordic Journal of Botany 25(5–6): 303–305. 10.1111/j.0107-055X.2008.00198.x

